# Author Correction: Associative learning and extinction of conditioned threat predictors across sensory modalities

**DOI:** 10.1038/s42003-021-02542-y

**Published:** 2021-08-18

**Authors:** Laura. R. Koenen, Robert. J. Pawlik, Adriane Icenhour, Liubov Petrakova, Katarina Forkmann, Nina Theysohn, Harald Engler, Sigrid Elsenbruch

**Affiliations:** 1 Institute of Medical Psychology and Behavioral Immunobiology, Center for Translational Neuro- and Behavioral Sciences, University Hospital Essen, University of Duisburg-Essen, Essen, Germany; 2 Department of Medical Psychology and Medical Sociology, Faculty of Medicine, Ruhr University Bochum, Bochum, Germany; 3 Translational Pain Research Unit, Department of Neurology, University Hospital Essen, University of Duisburg-Essen, Essen, Germany; 4 Institute of Diagnostic and Interventional Radiology and Neuroradiology, University Hospital Essen, University of Duisburg-Essen, Essen, Germany

**Keywords:** Fear conditioning, Extinction, Classical conditioning, Human behaviour

Correction to: *Communications Biology* 10.1038/s42003-021-02008-1, published online 11 May 2021.

The original version of this Article contained an error in the spelling of the author Liubov Petrakova, which was incorrectly given as Ljubov Petrakova. This has now been corrected in both the PDF and HTML versions of the Article.

