# Supplementary material for: Associative learning and extinction of conditioned threat predictors across sensory modalities
Source: Commun Biol. 2021 May 11;4:553. doi: 10.1038/s42003-021-02008-1 (PMC8113515; doi:10.1038/s42003-021-02008-1)
Supplement: Supplementary file 2 — Supplementary information [file 42003_2021_2008_MOESM2_ESM.pdf]

SUPPLEMENTARY INFORMATION

Associative learning and extinction of conditioned threat predictors across sensory modalities

Laura. R. Koenen[1], Robert. J. Pawlik[2], Adriane Icenhour[3], Ljubov Petrakova[2], Katarina Forkmann[3], Nina Theysohn[4], Harald Engler[1], Sigrid Elsenbruch[2,3*]

[1]Institute of Medical Psychology and Behavioral Immunobiology, University Hospital Essen, University of Duisburg-Essen, Hufelandstraße 55, 45147 Essen, Germany

[2]Department of Medical Psychology and Medical Sociology, Faculty of Medicine, Ruhr University Bochum, Universitätsstraße 150, D-44801 Bochum, Germany

[3]Translational Pain Research Unit, Department of Neurology, University Hospital Essen, University of Duisburg-Essen, Hufelandstraße 55, 45147 Essen, Germany

[4]Institute of Diagnostic and Interventional Radiology and Neuroradiology, University Hospital Essen, University of Duisburg-Essen, Hufelandstraße 55, 45147 Essen, Germany

*sigrid.elsenbruch@ruhr-uni-bochum.de

SUPPLEMENTARY MATERIALS

Table of contents

Supplementary methods..4

Details on thresholding and matching of unconditioned stimuli (US) 4

Supplementary tables 5

Table S1: Differences in neural activation induced by interoceptive versus exteroceptive threats implemented as unconditioned stimuli (US) during acquisition with US unpleasantness as covariate of no interest 5

Table S2: Differences in neural activation induced by interoceptive versus exteroceptive threats implemented as unconditioned stimuli (US) during acquisition with US intensity as covariate of no interest in study 1 7

Table S3: Shared neural activation induced by interoceptive and exteroceptive threats implemented as unconditioned stimuli (US) during acquisition in studies 1 and 2 8

Table S4: Results of rmANOVA of differential CS valence in study 1 9

Table S5: Results of rmANOVA of differential CS valence in study 2 10

Table S6: Results of paired t-tests of differential CS valence in study 1 11

Table S7: Results of paired t-tests of differential CS valence in study 2 12

Table S8: Shared neural activation induced by cues (CS) predicting interoceptive versus exteroceptive threats during acquisition in studies 1 and 2 13

Table S9: Differences in neural activation induced by cues (CS) predicting interoceptive versus exteroceptive threats during acquisition with US unpleasantness as covariate of no interest 15

Table S10: Differences in neural activation induced by cues (CS) predicting interoceptive versus exteroceptive threats during acquisition with US intensity as covariate of no interest in study 1...16

Table S11: Shared neural activation induced by cues (CS) predicting interoceptive versus exteroceptive threats during extinction in studies 1 and 2..17

Table S12: Differences in neural activation induced by cues (CS) predicting interoceptive versus exteroceptive threats during extinction..19

Supplementary figures 20

Figure S1: Exploratory analyses of skin conductance responses to conditioned predictors of interoceptive and exteroceptive threats for a subset of participants 20

Figure S2: Shared neural activation induced by cues (CS) predicting interoceptive versus exteroceptive threats during extinction in studies 1 and 2..21

Figure S3: Head movement parameters in pain trials during habituation phase in study 1.............22

Figure S4: Results of whole-brain analyses of neural activation induced by interoceptive versus exteroceptive threats implemented as unconditioned stimuli (US) during acquisition in studies 1 and 2. 23

Figure S5: Results of whole-brain analyses of neural activation induced by cues (CS) predicting interoceptive and exteroceptive threats during acquisition in studies 1 and 2. 24

Figure S6: Results of whole-brain analyses of neural activation induced by cues (CS) predicting interoceptive and exteroceptive threats during extinction in studies 1 and 2. 25

Figure S7: Results of whole-brain analyses of neural activation induced by cues (CS) predicting interoceptive and exteroceptive threats during reinstatement-test in studies 1 and 2..................26

Supplementary analyses 27
Supplementary references..28

Supplementary methods

Details on thresholding and matching of unconditioned stimuli (US)

All perceptual thresholds were assessed using established methods in our own prior work and in the field[1-8]. For visceral pain thresholds, assessed using pressure-controlled rectal distensions, a staircase ramp distention protocol with pressure increments of 5 mmHg was implemented in both studies. Perceptual ratings were obtained after each distension, and the procedure was terminated when participants indicated a distension pressure as "painful perception". Maximal rectal distension pressure was limited to 55 mmHg for safety reasons. For thermal stimuli applied in study 1, the mean pain threshold temperature of five consecutive measurements was calculated based on the quantitative sensory testing (QST) protocol[7]. Pain thresholds were obtained with ramped stimuli (1 °C/s) that were terminated when the volunteer pressed a response key. The baseline temperature was 32 °C (centre of neutral range). For the thermode, a temperature limit was set at 50 °C to avoid tissue damage[7]. For auditory stimuli applied in study 2, a series of five tones was presented with ascending tone volumes starting at 40 dB until volunteers pressed a response key indicating first sensation of unpleasantness. The unpleasantness threshold was set as the maximal volume determined in the final of the five series. A maximum loudness level of 120 dB was never exceeded.

As part of matching of US to perceived intensity (study 1) or perceived unpleasantness (study 2), durations of ascending and plateau phases of US were adjusted individually to overlap as closely as possible for each participant. This was done given small inter-individual differences in the time needed by the barostat system (with a preset pumping speed of air volume per second) to fully inflate the rectal balloon to the required individual pressure as calibrated during the thresholding procedure (i.e., slightly longer time required in individuals with a high rectal pain threshold). Hence, settings of the thermode for speed of heating/cooling (study 1) and of the sound system for increase/decrease of loudness (study 2) were adjusted individually to achieve matching US. This resulted in small inter-individual differences in durations of ascending (inflation/heating/increase of loudness) and plateau phases of US. In order to achieve comparable stimulus lengths, ascending and plateau phases were combined for analyses of functional magnetic resonance imaging (fMRI) data, resulting in identical predefined US durations set for analysis of BOLD response (20 s in study 1 and 14 s in study 2).

Supplementary tables

Table S1: Differences in neural activation induced by interoceptive versus exteroceptive threats implemented as unconditioned stimuli (US) during acquisition with US unpleasantness as covariate of no interest

		MNI-Coordinates					
**Contrast**	**Region**	**H**	**x**	**y**	**z**	**t**	***P***
**Study 1** (N=42)							
$US_{VISC} > US_{SOM}$							
ROI analyses	aINS	L	-34	6	12	7.18	<0.001
		R	34	14	8	5.39	<0.001
	dACC	L	-8	12	38	4.78	0.001
	amygdala	L	-24	-4	-12	3.44	0.018
Whole-brain analyses	*PCC*	*R*	*4*	*-40*	*12*	*3.82*	*<0.001*
	inferior frontal gyrus, opercular (dlPFC)	*R*	*38*	*8*	*32*	*3.83*	*<0.001*
	supramarginal gyrus (S2)	*L*	*-62*	*-26*	*24*	*4.15*	*<0.001*
		R	*60*	*-24*	*28*	*3.71*	*<0.001*
	vermis	*-*	*0*	*-54*	*-36*	*5.69*	*<0.001*
	cerebellum	*R*	*32*	*-52*	*-30*	*4.14*	*<0.001*
$US_{VISC} < US_{SOM}$							
ROI analyses	pINS	R	38	-12	18	6.26	<0.001
Whole-brain analyses	*superior frontal gyrus, medial*	*L*	*-10*	*48*	*34*	*3.68*	*<0.001*
	PHIP	*R*	*20*	*-6*	*-22*	*4.12*	*<0.001*
	postcentral (S1)	*L*	*-54*	*-12*	*48*	*4.03*	*<0.001*
		R	*44*	*-22*	*54*	*3.61*	*<0.001*
	postcentral (S2)	*L*	*-62*	*-6*	*30*	*3.72*	*<0.001*
		R	*64*	*-4*	*24*	*5.35*	*<0.001*
	SMA	*R*	*8*	*-20*	*68*	*5.41*	*<0.001*
	fusiform gyrus	*R*	*36*	*-44*	*-10*	*4.38*	*<0.001*
	rolandic operculum	*L*	*-44*	*-14*	*18*	*4.22*	*<0.001*
	middle temporal gyrus	*L*	*-62*	*-8*	*-14*	*5.07*	*<0.001*
		R	*60*	*-8*	*-16*	*3.69*	*0.001*
**Study 2** (N=23)							
$US_{VISC} > US_{AUD}$							
ROI	aINS	L	-44	2	8	4.72	0.005
		R	36	8	12	5.58	0.001
	dACC	R	2	8	40	5.12	0.002
Whole-brain analyses	*SMA*	*R*	*6*	*2*	*46*	*6.19*	*<0.001*
	rolandic operculum	*L*	*-46*	*2*	*8*	*5.31*	*<0.001*
$US_{VISC} < US_{AUD}$							
ROI	pINS	L	-38	-12	18	5.20	0.004
		R	38	-10	16	5.56	0.002
Whole-brain analyses	*inferior temporal gyrus*	*L*	*-52*	*-58*	*-6*	*4.49*	*<0.001*
	middle temporal gyrus	*R*	*56*	*-36*	*6*	*9.65*	*<0.001*
	superior temporal gyrus	*L*	*-54*	*-36*	*10*	*7.48*	*<0.001*
	rolandic operculum	*R*	*44*	*-8*	*24*	*6.74*	*<0.001*
	lingual area	*L*	*-16*	*-46*	*-8*	*4.30*	*<0.001*

	R	*20*	*-52*	*-10*	*5.48*	*<0.001*
angular gyrus	*L*	*-48*	*-52*	*24*	*4.64*	*<0.001*
precuneus	*R*	*6*	*-52*	*44*	*4.10*	*<0.001*
superior occipital gyrus	*L*	*-24*	*-76*	*24*	*4.30*	*<0.001*
middle occipital gyrus	*L*	*-44*	*-76*	*8*	*4.01*	*<0.001*

Differential neural activation induced by interoceptive threat (US_{VISC}) versus exteroceptive threat (study 1, US_{SOM}; study 2, US_{AUD}) implemented as unconditioned stimuli (US) during acquisition (for analyses without covariate, cf. Table 1), controlling for differences in US unpleasantness ratings assessed after acquisition as covariate of no interest. Peak voxel indicate results of ROI-analyses (cluster size $k_E \geq 3$; all P_{FWE}<0.05) and whole-brain analyses (*in italic font*; cluster size $k_E \geq 10$; all $P_{uncorrected}$<0.001). Exact unilateral *P*-values are provided.

Abbreviations: aINS, anterior insula; AUD, auditory; dACC, dorsal anterior cingulate cortex; dlPFC, dorsolateral prefrontal cortex; FWE, family-wise error; H, hemisphere; MNI, Montreal Neurological Institute; PCC, posterior cingulate cortex; PHIP, parahippocampus; pINS, posterior insula; ROI, regions of interest; S1, primary somatosensory cortex; S2, secondary somatosensory cortex; SMA, supplementary motor area; US, unconditioned stimuli; VISC, visceral.

Table S2: Differences in neural activation induced by interoceptive versus exteroceptive threats implemented as unconditioned stimuli (US) during acquisition with US intensity as covariate of no interest in study 1

		MNI-Coordinates					
**Contrast**	**Region**	**H**	**x**	**y**	**z**	**t**	***P***
**Study 1** (N=42)							
$US_{VISC} > US_{SOM}$							
ROI analyses	aINS	L	-34	6	12	7.79	<0.001
		R	34	14	8	5.40	0.001
	dACC	L	-8	12	38	4.64	0.001
	amygdala	L	-24	-4	-12	3.52	0.015
Whole-brain analyses	*PCC*	*R*	*4*	*-40*	*12*	*3.86*	*<0.001*
	inferior frontal gyrus, opercular (dlPFC)	*R*	*38*	*8*	*32*	*4.09*	*<0.001*
	superior frontal gyrus, orbital (vlPFC)	*R*	*20*	*58*	*-10*	*4.06*	*<0.001*
	supramarginal gyrus (S2)	*L*	*-62*	*-26*	*24*	*4.08*	*<0.001*
		R	*66*	*-28*	*28*	*3.66*	*<0.001*
	vermis	*-*	*0*	*-54*	*-36*	*5.76*	*<0.001*
	cerebellum	*R*	*32*	*-52*	*-30*	*4.13*	*<0.001*
$US_{VISC} < US_{SOM}$							
ROI analyses	pINS	R	38	-12	18	6.54	<0.001
Whole-brain analyses	*superior frontal gyrus, medial (dmPFC)*	*L*	*-10*	*48*	*34*	*3.71*	*<0.001*
	PHIP	*R*	*20*	*-6*	*-22*	*4.16*	*<0.001*
	postcentral gyrus (S1)	*L*	*-54*	*-12*	*48*	*4.04*	*<0.001*
		R	*44*	*-22*	*54*	*3.71*	*0.001*
	postcentral gyrus (S2)	*L*	*-62*	*-6*	*30*	*3.79*	*<0.001*
		R	*64*	*-4*	*24*	*5.45*	*<0.001*
	SMA	*R*	*8*	*-20*	*68*	*5.43*	*<0.001*
	fusiform gyrus	*R*	*36*	*-44*	*-10*	*4.40*	*<0.001*
	rolandic operculum	*L*	*-44*	*-14*	*18*	*4.34*	*<0.001*
	middle temporal gyrus	*L*	*-62*	*-8*	*-14*	*5.01*	*<0.001*
		R	*60*	*-8*	*-16*	*3.54*	*0.001*

Differential neural activation induced by interoceptive threat (US_{VISC}) versus exteroceptive threat (US_{SOM}) implemented as unconditioned stimuli (US) during acquisition (for analyses without covariate, cf. Table 1), controlling for differences in US intensity ratings assessed after acquisition in study 1 as covariate of no interest. Peak voxel indicate results of ROI-analyses (cluster size $k_E \geq 3$; all $P_{FWE}<0.05$) and whole-brain analyses (*in italic font*; cluster size $k_E \geq 10$; all $P_{uncorrected}<0.001$). Exact unilateral *P*-values are provided.

Abbreviations: aINS, anterior insula; dACC, dorsal anterior cingulate cortex; dmPFC, dorsomedial prefrontal cortex; dlPFC, dorsolateral prefrontal cortex; FWE, family-wise error; H, hemisphere; MNI, Montreal Neurological Institute; PCC, posterior cingulate cortex; PHIP, parahippocampus; pINS, posterior insula; ROI, regions of interest; S1, primary somatosensory cortex; S2, secondary somatosensory cortex; SMA, supplementary motor area; SOM, somatic; US, unconditioned stimuli; vlPFC, ventrolateral prefrontal cortex; VISC, visceral.

Table S3: Shared neural activation induced by interoceptive and exteroceptive threats implemented as unconditioned stimuli (US) during acquisition in studies 1 and 2

		MNI-Coordinates					
**Contrast**	**Region**	**H**	**x**	**y**	**z**	**t**	***P***
**Study 1** (N=42)							
$US_{VISC} \cap US_{SOM}$							
ROI analyses	aINS	L	-30	18	2	2.83	0.003
		R	44	18	0	4.25	<0.001
Whole-brain analyses	*MCC*	*L*	*-2*	*-24*	*32*	*2.26*	*<0.001*
	caudate nucleus	*R*	*10*	*14*	*10*	*2.63*	*<0.001*
	middle frontal gyrus (dlPFC)	*L**	*-36*	*12*	*40*	*4.37*	*<0.001*
		*R**	*40*	*18*	*42*	*3.83*	*<0.001*
	inferior frontal gyrus, orbital (vmPFC)	*L*	*-22*	*26*	*-12*	*2.27*	*<0.001*
	inferior frontal gyrus, opercular (vlPFC)	*R**	*52*	*12*	*14*	*4.72*	*<0.001*
	inferior frontal gyrus, triangular (vlPFC)	*L**	*-46*	*44*	*2*	*4.11*	*<0.001*
		*R**	*46*	*46*	*-2*	*5.44*	*<0.001*
	superior frontal gyrus, medial (dmPFC)	*R**	*8*	*28*	*44*	*4.29*	*<0.001*
	midtemporal gyrus	*L*	*-54*	*-36*	*-12*	*2.46*	*<0.001*
		R	*62*	*-30*	*-6*	*2.31*	*<0.001*
	inferior parietal gyrus	*L**	*-52*	*-48*	*52*	*5.27*	*<0.001*
		*R**	*50*	*-56*	*48*	*5.01*	*<0.001*
	middle occipital gyrus	*L**	*-28*	*-92*	*0*	*9.84*	*<0.001*
	calcarine fissure	*R**	*26*	*-92*	*2*	*7.15*	*<0.001*
	cerebellum	*L**	*-32*	*-72*	*-38*	*3.94*	*<0.001*
		R	*14*	*-80*	*-28*	*2.49*	*<0.001*
**Study 2** (N=23)							
$US_{VISC} \cap US_{AUD}$							
Whole-brain analyses	*lingual gyrus*	*L*	*-18*	*80*	*-8*	*7.95*	*<0.001*
	superior temporal gyrus	*L*	*-52*	*-6*	*2*	*2.96*	*<0.001*
	supramarginal gyrus (S2)	*L*	*-60*	*-26*	*18*	*2.51*	*<0.001*
		R	*50*	*-32*	*24*	*2.23*	*<0.001*

Shared neural activation induced by interoceptive (US_{VISC}) versus exteroceptive threat (study 1, US_{SOM}; study 2, US_{AUD}) during acquisition. Results of conjunction analyses against global null are presented (*Results also significant against conjunction null). Peak voxels indicate results of ROI-analyses (all P_{FWE}<0.05) and whole-brain analyses (*in italic font*; cluster size $k_E \geq 10$; all $P_{uncorrected}$<0.001). Exact unilateral *P*-values are provided. For analysis of differential responses, see Fig. 1, Table 1.

Abbreviations: AUD, auditory; dlPFC, dorsolateral prefrontal cortex; dmPFC, dorsomedial prefrontal cortex; H, hemisphere; MCC, midcingulate cortex; MNI, Montreal Neurological Institute; ROI, regions of interest; SOM, somatic; US, unconditioned stimuli; VISC, visceral; vlPFC, dorsolateral prefrontal cortex; vmPFC, dorsomedial prefrontal cortex.

Table S4: Results of rmANOVA of differential CS valence in study 1

Phase	Effects	df	F	*P*	η_p^2
ACQ	Time	1,41	28.42	**<0.001**	0.41
	Modality	1,41	9.64	**0.003**	0.19
	Time x Modality	1,41	16.71	**<0.001**	0.29
EXT	Time	1,41	6.38	**0.015**	0.14
	Modality	1,41	16.26	**<0.001**	0.28
	Time x Modality	1,41	5.51	**0.024**	0.12
RST-TEST	Time	1,40	1.49	0.229	0.04
	Time x Group	1,40	2.78	0.104	0.07
	Modality	1,40	10.45	**0.002**	0.21
	Modality x Group	1,40	0.04	0.847	<0.01
	Group	1,40	0.81	0.374	0.02
	Time x Modality	1,40	6.10	**0.018**	0.13
	Time x Modality x Group	1,1,40	5.56	**0.023**	0.12

Results of 2x2 repeated measure analyses of variance (rmANOVA) of differential CS valence in study 1 for the acquisition (ACQ, N=42), extinction (EXT, N=42), and reinstatement-test (RST-TEST) phases after single threat reinstatement with one US modality (US_{VISC}-subgroup: N=22; US_{SOM}-subgroup: N=20). RmANOVA were computed with the within-factors time (pre, post) and modality (CS type: interoceptive, exteroceptive). For a visualization of results, see Fig. 2 and 3.

Abbreviations: CS, conditioned stimuli; SOM, somatic; US, unconditioned stimuli; VISC, visceral.

Table S5: Results of rmANOVA of differential CS valence in study 2

Phase	Effects	df	F	*P*	η_p^2
ACQ	Time	1,22	23.05	**<0.001**	0.51
	Modality	1,22	2.05	0.167	0.09
	Time x Modality	1,22	8.19	**0.009**	0.27
EXT	Time	1,22	10.73	**0.003**	0.33
	Modality	1,22	3.05	0.095	0.12
	Time x Modality	1,22	6.36	**0.019**	0.22
RST-TEST	Time	1,22	2.73	0.112	0.11
	Modality	1,22	3.20	0.087	0.13
	Time x Modality	1,22	4.12	0.055	0.16

Results of 2x2 repeated measure analyses of variance (rmANOVA) of differential CS valence in study 2 (N=23) for the acquisition (ACQ), extinction (EXT), and reinstatement-test (RST-TEST) phases after multiple threat reinstatement with both US modalities (US_{VISC}+US_{AUD}). RmANOVA were computed with the within-factors time (pre, post) and modality (CS type: interoceptive, exteroceptive). For a visualization of results, see Fig. 2 and 3. Abbreviations: AUD, auditory; CS, conditioned stimuli; US, unconditioned stimuli; VISC, visceral.

Table S6: Results of paired t-tests of differential CS valence in study 1

Phase	Paired t-test	t	df	*P*	d
ACQ	Within-modality (PRE-POST)				
	ΔCS^{+}_{VISC}	-5.92	41	**<0.001**	1.20
	ΔCS^{+}_{SOM}	-3.12	41	**0.003**	0.54
	Between-modality (ΔCS^{+}_{VISC} - ΔCS^{+}_{SOM})				
	PRE	-0.35	41	0.727	0.05
	POST	4.19	41	**<0.001**	0.59
EXT	Within-modality (PRE-POST)				
	ΔCS^{+}_{VISC}	3.05	41	**0.004**	0.38
	ΔCS^{+}_{SOM}	1.62	41	0.112	0.22
	Between-modality (ΔCS^{+}_{VISC} - ΔCS^{+}_{SOM})				
	PRE (same as POST ACQ, see above)			-	
	POST	3.42	41	**0.001**	0.57
RST-TEST					
US_{VISC}-subgroup	Within-modality (PRE-POST)				
	ΔCS^{+}_{VISC}	-0.28	21	0.786	0.03
	ΔCS^{+}_{SOM}	-0.27	21	0.787	0.04
	Between-modality (ΔCS^{+}_{VISC} - ΔCS^{+}_{SOM})				
	PRE	2.29	21	**0.032**	0.50
	POST	2.73	21	**0.013**	0.58
US_{SOM}-subgroup	Within-modality (PRE-POST)				
	ΔCS^{+}_{VISC}	3.15	19	**0.005**	0.23
	ΔCS^{+}_{SOM}	0.62	19	0.544	0.04
	Between-modality (ΔCS^{+}_{VISC} - ΔCS^{+}_{SOM})				
	PRE	2.48	19	**0.023**	0.52
	POST	1.40	19	0.177	0.21

Results of paired t-tests of differential CS valence in study 1 testing differences between time points (PRE-POST) within-modalities, and differences between-modalities (ΔCS^{+}_{VISC} vs. ΔCS^{+}_{SOM}) for specific time points for the acquisition (ACQ, N=42), extinction (EXT, N=42), and reinstatement-test (RST-TEST) phases after single threat reinstatement with one US modality (US_{VISC}-subgroup: N=22; US_{SOM}-subgroup: N=20). All *P*-values are uncorrected. For a visualization of results, see Fig. 2 and 3.

Abbreviations: CS, conditioned stimuli; SOM, somatic; US, unconditioned stimuli; VISC, visceral.

Table S7: Results of paired t-tests of differential CS valence in study 2

Phase	Paired t-test	t	df	*P*	d
ACQ	Within-modality (PRE-POST)				
	ΔCS^{+}_{VISC}	-5.48	22	**<0.001**	1.36
	ΔCS^{+}_{AUD}	-3.64	22	**0.001**	0.82
	Between-modality (ΔCS^{+}_{VISC} - ΔCS^{+}_{AUD})				
	PRE	-1.19	22	0.245	0.21
	POST	0.28	22	**0.003**	0.37
EXT	Within-modality (PRE-POST)				
	ΔCS^{+}_{VISC}	4.31	22	**<0.001**	1.00
	ΔCS^{+}_{AUD}	2.07	22	0.050	0.60
	Between-modality (ΔCS^{+}_{VISC} - ΔCS^{+}_{AUD})				
	PRE	2.32	22	**0.030**	0.40
	MID*	0.44	22	**0.678**	0.40
	POST	0.44	22	0.662	0.12
RST	Within-modality (PRE-POST)				
	ΔCS^{+}_{VISC}	-2.20	22	**0.038**	0.50
	ΔCS^{+}_{AUD}	-0.09	22	0.929	0.02
	Between-modality (ΔCS^{+}_{VISC} - ΔCS^{+}_{AUD})				
	PRE (same as POST EXT, see above)			-	
	POST	2.49	22	**0.021**	0.73

Results of paired t-tests of differential CS valence in study 2 (N=23) testing differences between time points (PRE-POST) within-modalities, and differences between-modalities (ΔCS^{+}_{VISC} vs. ΔCS^{+}_{AUD}) for specific time points for the acquisition (ACQ), extinction (EXT), and reinstatement-test (RST-TEST) phases after multiple threat reinstatement with both US modalities (US_{VISC}+US_{AUD}). *Additional VAS mid-extinction after 6 trials per CS type. All *P*-values are uncorrected. For a visualization of results, see Fig. 2 and 3.

Abbreviations: AUD, auditory; CS, conditioned stimuli; US, unconditioned stimuli; VISC, visceral.

Table S8: Shared neural activation induced by cues (CS) predicting interoceptive versus exteroceptive threats during acquisition in studies 1 and 2

		MNI-Coordinates					
**Contrast**	**Region**	**H**	**x**	**y**	**z**	**t**	***P***
**Study 1** (N=42)							
$\Delta CS^{+}_{VISC} \cap \Delta CS^{+}_{SOM}$							
ROI analyses[1]	aINS	R	32	30	6	2.60	0.008
	HIP	R	34	-38	-2	4.08	<0.001
Whole-brain analyses[1]	*middle occipital gyrus*	*R*	*34*	*-90*	*10*	*3.55*	*<0.001*
	cerebellum	*R*	*24*	*-66*	*-46*	*2.47*	*<0.001*
ROI analyses[2]	aINS	L	-30	8	-14	3.13	<0.001
		R	36	12	-12	3.30	<0.001
	MCC	L*	-2	-6	38	4.55	0.002
	dACC	R	0	18	28	3.22	<0.001
	amygdala	L	-24	2	-24	2.39	0.007
		R	22	6	-16	2.54	0.004
Whole-brain analyses[2]	*sgACC*	*R*	*2*	*32*	*-6*	*3.13*	*<0.001*
	superior frontal gyrus, medial (dmPFC)	*R*	*8*	*52*	*2*	*3.07*	*<0.001*
	inferior frontal gyrus, orbital (vlPFC)	*R*	*36*	*30*	*-16*	*2.55*	*<0.001*
	rolandic operculum	*R*	*-46*	*-4*	*12*	*3.22*	*<0.001*
	cuneus	*R*	*12*	*-86*	*20*	*3.29*	*<0.001*
	caudat nucleus	*R*	*14*	*10*	*16*	*2.65*	*<0.001*
	midtemporal pole	*L*	*-48*	*10*	*-34*	*2.73*	*<0.001*
		R	*52*	*-2*	*-20*	*4.00*	*<0.001*
	superior temporal gyrus	*L*	*-54*	*-32*	*16*	*2.50*	*<0.001*
	inferior temporal gyrus	*L**	*-42*	*-16*	*-24*	*3.77*	*<0.001*
	superior parietal gyrus	*R**	*24*	*-54*	*70*	*4.70*	*<0.001*
	vermis	*R*	*2*	*-36*	*-8*	*3.44*	*<0.001*
	cerebellum	*L**	*-22*	*-26*	*-28*	*5.68*	*<0.001*
		R	*12*	*-40*	*-34*	*3.16*	*<0.001*
**Study 2** (N=23)							
$\Delta CS^{+}_{VISC} \cap \Delta CS^{+}_{AUD}$							
ROI analyses	aINS	L	-42	0	0	2.86	0.002
	MCC	R	4	-4	38	2.88	0.002
	dACC	R	2	16	32	3.84	<0.001
	HIP	L	-12	36	0	3.51	<0.001
		R	12	-34	8	3.11	0.001
Whole-brain analyses	*lingual*	*L*	*-10*	*-38*	*-2*	*4.30*	*<0.001*
	precuneus	*L*	*-16*	*-56*	*34*	*4.11*	*<0.001*
	cuneus	*L*	*-8*	*-80*	*16*	*4.58*	*<0.001*
	putamen	*R*	*32*	*-8*	*4*	*3.59*	*<0.001*
	SMA	*L**	*-12*	*4*	*46*	*6.32*	*<0.001*
	rolandic operculum	*L*	*-44*	*-4*	*6*	*3.28*	*<0.001*
	postcentral	*L*	*-50*	*-12*	*34*	*2.29*	*<0.001*
		R	*42*	*-22*	*34*	*2.61*	*<0.001*
	middle occipital gyrus	*L**	*-44*	*-74*	*12*	*4.73*	*<0.001*
	vermis	*-**	*0*	*-48*	*-18*	*7.62*	*<0.001*

Shared neural activation induced by predictors of interoceptive (CS^+_{VISC}) versus exteroceptive threat (study 1, CS^+_{SOM}; study 2, CS^+_{AUD}) relative to safety-predictive CS-, during acquisition. Results of conjunction analysis against global null are presented (study 1: [1]$\{CS^+_{VISC} > CS^-\} \cap \{CS^+_{SOM} > CS^-\}$; [2]$\{CS^+_{VISC} < CS^-\} \cap \{CS^+_{SOM} < CS^-\}$; study 2: all analyses based on $\{CS^+_{VISC} < CS^-\} \cap \{CS^+_{AUD} < CS^-\}$; *Results also significant against conjunction null). Peak voxel indicate results of ROI-analyses (cluster size $k_E \geq 3$; all $P_{FWE}<0.05$) and whole-brain analyses (*in italic font*; cluster size $k_E \geq 10$; all $P_{uncorrected}<0.001$). Exact unilateral *P*-values are provided. For analysis of differential responses, see Fig.2, Table 3.

Abbreviations: aINS, anterior insula; AUD, auditory; CS, conditioned stimuli; dACC, dorsal anterior cingulate cortex; dmPFC, dorsomedial prefrontal cortex; FWE, family-wise error; H, hemisphere; HIP, hippocampus; MCC, midcingulate cortex; MNI, Montreal Neurological Institute; ROI, regions of interest; sgACC, subgenual anterior cingulate cortex; SMA, supplementary motor area; SOM, somatic; VISC, visceral; vlPFC, ventrolateral prefrontal cortex.

Table S9: Differences in neural activation induced by cues (CS) predicting interoceptive versus exteroceptive threats during acquisition with US unpleasantness as covariate of no interest

		MNI-Coordinates					
**Contrast**	**Region**	**H**	**x**	**y**	**z**	**t**	***P***
**Study 1** (N=42)							
$CS^{+}_{VISC} < CS^{+}_{SOM}$							
ROI analyses	pINS	L	-36	-8	18	4.29	0.008
		R	32	-22	12	4.87	0.002
	MCC	R	6	-6	40	3.52	0.026
Whole-brain analyses	*superior frontal gyrus, medial (vmPFC)*	*R*	*10*	*60*	*-2*	*3.70*	*<0.001*
	precentral gyrus (S1)	*R*	*14*	*-30*	*74*	*5.56*	*<0.001*
	rolandic operculum	*L*	*-42*	*-30*	*16*	*3.51*	*0.001*
	thalamus	*L*	*-16*	*-22*	*2*	*3.76*	*<0.001*
		R	*14*	*-22*	*0*	*4.12*	*<0.001*
	putamen	*R*	*28*	*-8*	*14*	*5.21*	*<0.001*
	inferior temporal gyrus	*L*	*-58*	*-50*	*-14*	*3.63*	*<0.001*
	middle occipital lobe	*L*	*-32*	*-80*	*40*	*4.19*	*<0.001*
	vermis	*R*	*4*	*-44*	*-16*	*4.08*	*<0.001*
	cerebellum	*L*	*-36*	*-74*	*-34*	*4.25*	*<0.001*
		R	*36*	*-78*	*-26*	*4.78*	*<0.001*
$CS^{+}_{VISC} > CS^{+}_{SOM}$	-	-	-	-	-	-	-
**Study 2** (N=23)							
$CS^{+}_{VISC} < CS^{+}_{AUD}$							
ROI analyses	pINS	L	-34	-20	18	4.77	0.009
		R	44	-14	14	4.69	0.010
	MCC	R	6	-10	42	4.77	0.004
	dACC	R	4	6	36	4.09	0.016
Whole-brain analyses	*superior temporal gyrus*	*L*	*-42*	*-28*	*8*	*5.93*	*<0.001*
		R	*50*	*-22*	*10*	*6.60*	*<0.001*
$CS^{+}_{VISC} > CS^{+}_{AUD}$	-	-	-	-	-	-	-

Differential neural activation induced by predictors of interoceptive (CS^{+}_{VISC}) versus exteroceptive threat (study 1, CS^{+}_{SOM}; study 2, CS^{+}_{AUD}), during acquisition (for analyses without covariate, cf. Table 3), controlling for differences in US unpleasantness ratings assessed after acquisition as covariate of no interest. Peak voxel indicate results of ROI-analyses (cluster size $k_E \geq 3$; all $P_{FWE}<0.05$) and whole-brain analyses (*in italic font*; cluster size $k_E \geq 10$; all $P_{uncorrected}<0.001$). Exact unilateral *P*-values are provided.

Abbreviations: AUD, auditory; CS, conditioned stimuli; dACC, dorsal anterior cingulate cortex; FWE, family-wise error; H, hemisphere; MCC, midcingulate cortex; MNI, Montreal Neurological Institute; pINS, posterior insula; ROI, regions of interest; S1, primary somatosensory cortex; SOM, somatic; US, unconditioned stimuli; VISC, visceral; vmPFC, ventromedial prefrontal cortex.

Table S10: Differences in neural activation induced by cues (CS) predicting interoceptive versus exteroceptive threats during acquisition with US intensity as covariate of no interest in study 1

		MNI-Coordinates					
**Contrast**	**Region**	**H**	**x**	**y**	**z**	**t**	***P***
**Study 1** (N=42)							
$\Delta CS^{+}_{VISC} > \Delta CS^{+}_{SOM}$							
ROI analyses	pINS	L	-36	-8	18	4.28	0.009
		R	32	-22	12	4.84	0.002
	MCC	R	6	-6	40	3.52	0.026
Whole-brain analyses	*precentral gyrus (S1)*	*R*	*14*	*-30*	*74*	*5.51*	*<0.001*
	thalamus	*L*	*-16*	*-22*	*2*	*3.79*	*<0.001*
		R	*14*	*-22*	*0*	*4.12*	*<0.001*
	putamen	*R*	*28*	*-8*	*14*	*5.28*	*<0.001*
	inferior temporal gyrus	*L*	*-58*	*-48*	*-14*	*3.64*	*<0.001*
	middle occipital lobe	*L*	*-32*	*-80*	*38*	*4.24*	*<0.001*
	vermis	*R*	*4*	*-44*	*-16*	*4.07*	*<0.001*
	cerebellum	*L*	*-36*	*-74*	*-34*	*4.24*	*<0.001*
		R	*36*	*-78*	*-26*	*4.78*	*<0.001*
$\Delta CS^{+}_{VISC} < \Delta CS^{+}_{SOM}$	-	-	-	-	-	-	-

Differential neural activation induced by predictors of interoceptive (CS^{+}_{VISC}) versus exteroceptive threat (CS^{+}_{SOM}) relative to safety-predictive CS^{-}, during acquisition (for analyses without covariate, cf. Table 3), controlling for differences in US intensity ratings assessed after acquisition in study 1 as covariate of no interest. Peak voxel indicate results of ROI-analyses (cluster size $k_E \geq 3$; all $P_{FWE}<0.05$) and whole-brain analyses (*in italic font*; cluster size $k_E \geq 10$; all $P_{uncorrected}<0.001$). Exact unilateral *P*-values are provided. Abbreviations: AUD, auditory; CS, conditioned stimuli; FWE, family-wise error; H, hemisphere; MCC, midcingulate cortex; MNI, Montreal Neurological Institute; pINS, posterior insula; ROI, regions of interest; S1, primary somatosensory cortex; SOM, somatic; US, unconditioned stimuli; VISC, visceral.

Table S11: Shared neural activation induced by cues (CS) predicting interoceptive versus exteroceptive threats during extinction in studies 1 and 2

Contrast	Region	MNI-Coordinates				t	*P*
		H	x	y	z		
**Study 1** (N=42)							
$\Delta CS^{+}_{VISC} \cap \Delta CS^{+}_{SOM}$							
ROI analyses[1]	vmPFC	R	12	30	-20	3.03	0.001
	HIP	R	32	-36	0	4.38	<0.001
Whole-brain analyses[1]	*superior frontal gyrus (vlPFC)*	*L*	*-20*	*8*	*68*	*2.20*	*<0.001*
		R	*30*	*64*	*6*	*3.48*	*<0.001*
	superior frontal gyrus, orbital (vlPFC)	*L**	*-32*	*58*	*-2*	*4.12*	*<0.001*
	superior frontal gyrus, medial (dmPFC)	*R*	*4*	*52*	*46*	*3.10*	*<0.001*
	middle frontal gyrus (vlPFC)	*L**	*-34*	*50*	*-12*	*5.65*	*<0.001*
	middle frontal gyrus, orbital (vmPFC)	*L**	*-22*	*38*	*-10*	*3.90*	*<0.001*
	Inferior frontal gyrus, opercular (vlPFC)	*L**	*-44*	*12*	*12*	*2.84*	*<0.001*
	(vlPFC)	*R*	*34*	*10*	*34*	*3.99*	*<0.001*
	gyrus rectus (vmPFC)	*R*	*12*	*30*	*-20*	*3.03*	*<0.001*
	precentral	*L**	*-32*	*4*	*44*	*4.10*	*<0.001*
	postcentral	*L*	*-48*	*-34*	*52*	*2.56*	*<0.001*
	supramarginal gyrus (S2)	*L*	*-60*	*-52*	*32*	*3.44*	*<0.001*
	SMA	*R*	*4*	*24*	*66*	*2.40*	*<0.001*
	rectus	*L*	*-6*	*46*	*-24*	*2.58*	*<0.001*
	caudate	*L**	*-12*	*10*	*18*	*5.12*	*<0.001*
		*R**	*6*	*18*	*6*	*7.37*	*<0.001*
	inferior temporal gyrus	*L**	*-60*	*-28*	*-18*	*4.15*	*<0.001*
	midtemporal gyrus	*L*	*-60*	*-54*	*8*	*2.64*	*<0.001*
		*R**	*64*	*-46*	*8*	*4.09*	*<0.001*
	inferior parietal gyrus	*L**	*-26*	*-52*	*38*	*5.07*	*<0.001*
	middle occipital lobe	*L*	*-24*	*96*	*2*	*2.53*	*<0.001*
		R	*28*	*-94*	*8*	*2.71*	*<0.001*
	vermis	*R*	*6*	*-68*	*-10*	*3.06*	*<0.001*
	cerebellum	*L**	*-26*	*-58*	*-30*	*2.61*	*<0.001*
		*R**	*8*	*-60*	*-32*	*5.56*	*<0.001*
ROI analyses[2]	pINS	R	38	-28	20	3.51	<0.001
	amygdala	L	-24	-4	22	2.23	0.014
		R	28	0	-26	2.81	0.001
	HIP	L*	-22	-10	-26	4.30	<0.001
		R	26	-2	-26	2.57	0.014
Whole-brain analyses[2]	*PHIP*	*R*	*20*	*-4*	*-26*	*3.29*	*<0.001*
	postcentral (S1)	*L*	*-14*	*-44*	*76*	*2.76*	*<0.001*
		R	*24*	*-36*	*76*	*3.13*	*<0.001*
	supramarginal gyrus (S2)	*R*	*46*	*-36*	*30*	*2.58*	*<0.001*
	superior temporal gyrus	*R*	*60*	*-10*	*8*	*2.90*	*<0.001*
	heschl gyrus	*R**	*36*	*-32*	*12*	*4.74*	*<0.001*

	rolandic operculum	*L*	*-38*	*-28*	*20*	*2.86*	*<0.001*
	calcarine fissure	*L*	*-12*	*-54*	*4*	*2.65*	*<0.001*
	thalamus	*R*	*16*	*-24*	*-30*	*2.61*	*<0.001*
	precuneus	*-*	*0*	*-56*	*22*	*2.20*	*<0.001*
	cerebellum	*R*	*10*	*-34*	*-20*	*2.98*	*<0.001*
**Study 2** (N=23)							
$\Delta CS^{+}_{VISC} \cap \Delta CS^{+}_{AUD}$							
ROI analyses[1]	aINS	L	-46	2	-2	2.99	0.001
	dACC	L	-4	16	30	3.53	<0.001
	HIP	L*	-18	-36	10	4.24	<0.001
Whole-brain analyses[1]	*ACC*	*R*	*8*	*26*	*28*	*2.43*	*<0.001*
	PCC	*R*	*12*	*-48*	*30*	*2.52*	*<0.001*
	postcentral gyrus	R	*42*	*-28*	*36*	*4.62*	*<0.001*
	superior temporal gyrus	*L*	*-48*	*-36*	*16*	*2.84*	*<0.001*
		R	*50*	*-22*	*0*	*2.32*	*<0.001*
	putamen	*R*	*30*	*0*	*4*	*2.58*	*<0.001*
	thalamus	*R*	*8*	*-26*	*14*	*3.78*	*<0.001*
	angular gyrus	*L*	*-34*	*-56*	*36*	*2.40*	*<0.001*
	vermis	*R**	*4*	*-40*	*-20*	*4.30*	*<0.001*
	cerebellum	*R*	*8*	*-50*	*-6*	*2.51*	*<0.001*
ROI analyses[2]	front inf oper (dlPFC)	R	34	10	34	3.18	<0.001
Whole-brain analyses[2]	*front inf oper (dlPFC)*	*L*	*-40*	*2*	*22*	*2.51*	*<0.001*
	calcarine	*L*	*-6*	*-50*	*4*	*2.33*	*<0.001*
		*R**	*30*	*-54*	*10*	*3.75*	*<0.001*

Shared neural activation induced by predictors of interoceptive (CS^{+}_{VISC}) versus exteroceptive threat (study 1, CS^{+}_{SOM}; study 2, CS^{+}_{AUD}) relative to safety-predictive CS-, during extinction. Results of conjunction analysis against global null are presented (study 1: [1]$\{CS^{+}_{VISC} > CS^{-}\} \cap \{CS^{+}_{SOM} > CS^{-}\}$; [2]$\{CS^{+}_{VISC} < CS^{-}\} \cap \{CS^{+}_{SOM} < CS^{-}\}$; study 2: [1]$\{CS^{+}_{VISC} < CS^{-}\} \cap \{CS^{+}_{AUD} < CS^{-}\}$; [2]$\{CS^{+}_{VISC} > CS^{-}\} \cap \{CS^{+}_{AUD} > CS^{-}\}$; *Results also significant against conjunction null). Peak voxel indicate results of ROI-analyses (cluster size $k_E \geq 3$; all $P_{FWE}<0.05$) and whole-brain analyses (*in italic font*; cluster size $k_E \geq 10$; all $P_{uncorrected}<0.001$). Exact unilateral *P*-values are provided. For visualization of results, see supplementary Fig. S2 and S6.

Abbreviations: aINS, anterior insula; AUD, auditory; CS, conditioned stimuli; dACC, dorsal anterior cingulate cortex; dlPFC, dorsolateral prefrontal cortex; FWE, family-wise error; H, hemisphere; HIP, hippocampus; MNI, Montreal Neurological Institute; PCC, posterior cingulate cortex; pINS, posterior insula; ROI, regions of interest; S1, primary somatosensory cortex; S2, secondary somatosensory cortex; SOM, somatic; SMA, supplementary motor area; VISC, visceral; vlPFC, dorsolateral prefrontal cortex; vmPFC, dorsomedial prefrontal cortex.

Table S12: Differences in neural activation induced by cues (CS) predicting interoceptive versus exteroceptive threats during extinction

Contrast	Region	MNI-Coordinates H	x	y	z	t	*P*
**Study 1** (N=42)							
$\Delta CS^{+}_{VISC} > \Delta CS^{+}_{SOM}$	-	-	-	-	-	-	-
$\Delta CS^{+}_{VISC} < \Delta CS^{+}_{SOM}$	*middle temporal gyrus*	L	-36	-68	12	3.89	<0.001
	-	-	-	-	-	-	-
**Study 2** (N=23)							
$\Delta CS^{+}_{VISC} > \Delta CS^{+}_{AUD}$	-	-	-	-	-	-	-
$\Delta CS^{+}_{VISC} < \Delta CS^{+}_{AUD}$	-	-	-	-	-	-	-

Differential neural activation induced by predictors of interoceptive (CS^{+}_{VISC}) versus exteroceptive threat (study 1, CS^{+}_{SOM}; study 2, CS^{+}_{AUD}) relative to safety-predictive CS^{-}, during extinction. Results of second-level paired t-tests are presented (study 1: {CS^{+}_{VISC} < CS^{-}} > {CS^{+}_{SOM} < CS^{-}}; study 2: {CS^{+}_{VISC} < CS^{-}} > {CS^{+}_{AUD} < CS^{-}}; and vice versa). Peak voxel indicate results of whole-brain analyses (*in italic font*: cluster size $k_E \geq 10$; all $P_{uncorrected}$<0.001). No suprathreshold clusters were detectable for FWE-corrected ROI-analyses. Exact unilateral *P*-values are provided. For shared responses, see supplementary Fig. S2, Table S11. Abbreviations: AUD, auditory; CS, conditioned stimuli; FWE, family-wise error; H, hemisphere; MNI, Montreal Neurological Institute; ROI, regions of interest; SOM, somatic; VISC, visceral.

Supplementary figures

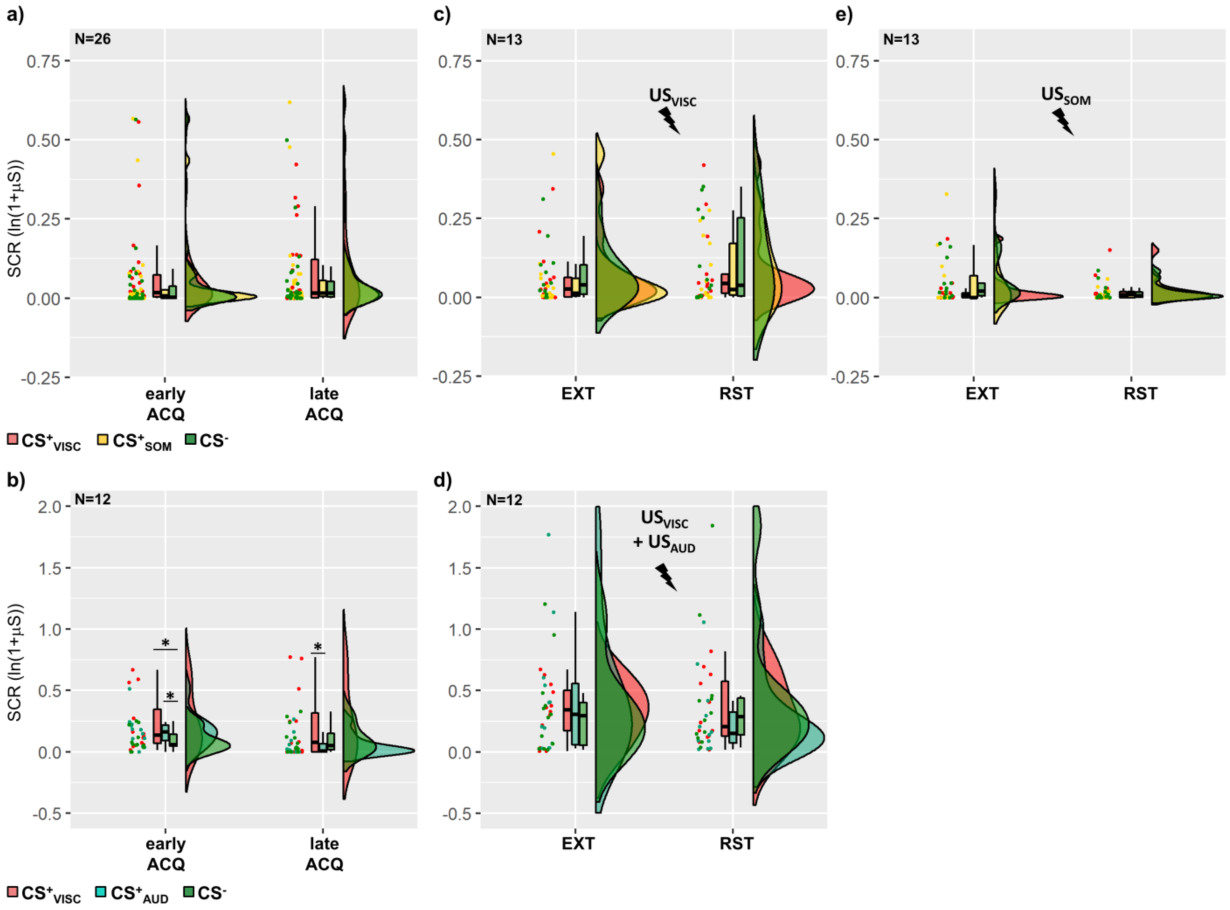

Figure S1: Exploratory analyses of skin conductance responses to conditioned predictors of interoceptive and exteroceptive threats for a subset of participants

Skin conductance responses (SCR) to conditioned predictors of interoceptive (CS^+_{VISC}) and exteroceptive threats (study 1, CS^+_{SOM}; study 2, CS^+_{AUD}) during acquisition (ACQ), extinction (EXT) and reinstatement-test (RST-TEST) in study 1 (upper row panels a,c,e) and study 2 (lower row panels b,d). Data in both studies was analysed in a subset of participants (N=26 in study 1, N=12 in study 2) after exclusion of incomplete datasets, participants with missing data and/or technical difficulties, and non-responders. Electrodermal activity (EDA) was recorded online from electrodes placed on the thenar and hypothenar of the left hand using an MRI-compatible system (Biopac Systems, Inc., Goleta, CA, USA; MP100 in study 1, MP160 in study 2). EDA data was processed using AcqKnowledge Software (Biopac; Version 3.9 in study1, Version 4.3 in study 2) and filtered for artefacts using a bandpass filter (10Hz - 0.05 Hz) SCR responses were obtained (1s after CS onset until US onset) with a SCR threshold of 0.01 microsiemens (μS)[9,10]. Note that comparability of data between studies is limited due to a newer data acquisition system being used in study 2, including an improved filter system, leading to a higher signal to noise ratio. All data was normalized using a log-transformation (ln(1+μS)).[9] Exploratory statistical analyses revealed greater SCR responses to CS^+_{VISC} when compared to CS^+_{AUD} and CS^- during the first (early) and second (late) half of trials in the acquisition phase of study 2 (*paired t-tests, all P<.05). No other significant differences for exploratory paired t-tests comparing CS^+_{VISC} and other CS were observed. Data is presented as individual data points, boxplots, and densities (raincloud plots[11]).

Abbreviations: ACQ, acquisition; AUD, auditory; CS, conditioned stimuli; EDA, electrodermal activity; EXT, extinction; MRI, magnetic resonance imaging; SCR, skin conductance response; RST-TEST, reinstatement-test; SOM, somatic; US, unconditioned stimuli; VISC, visceral.

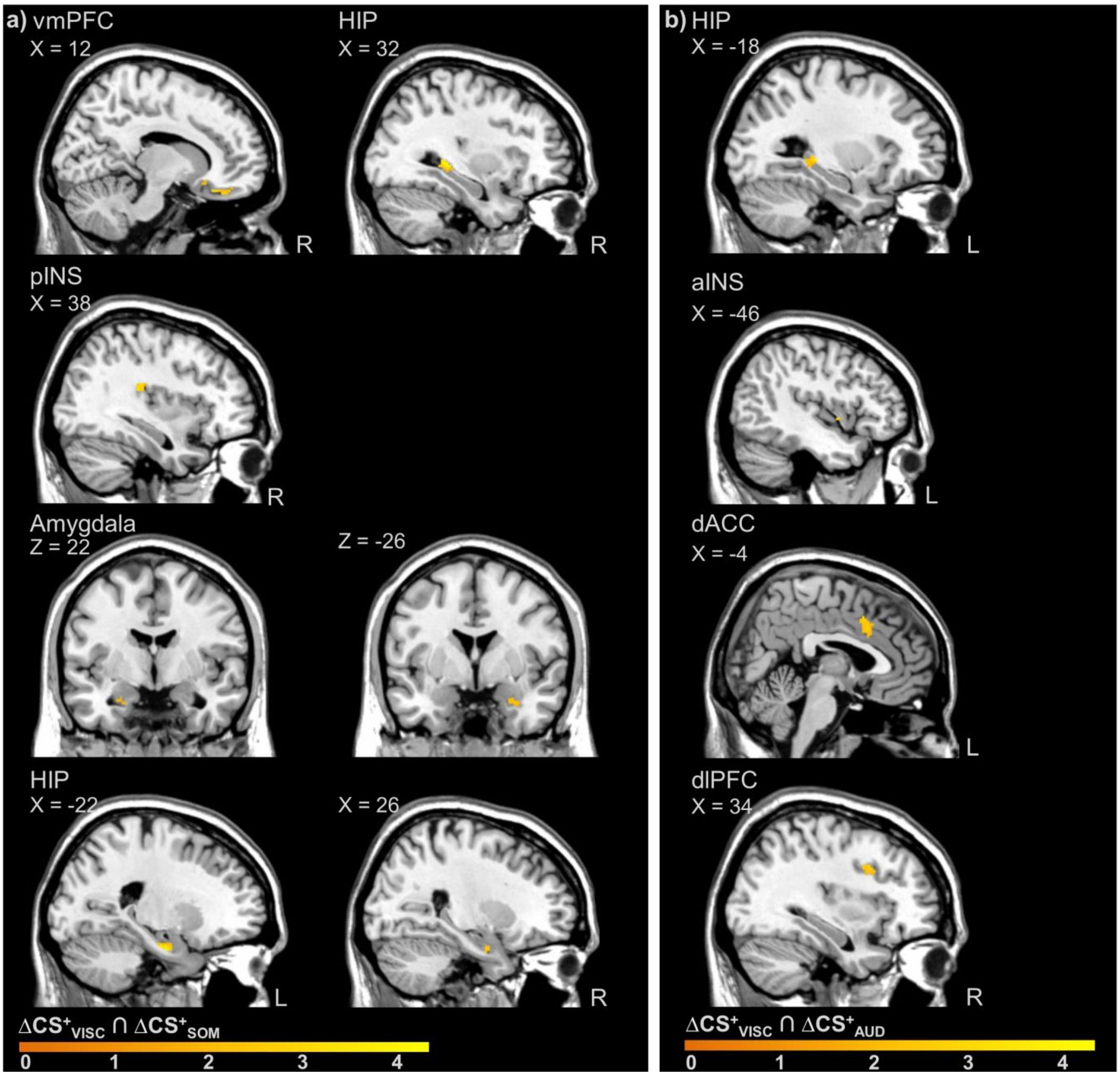

Figure S2: Shared neural activation induced by cues (CS) predicting interoceptive versus exteroceptive threats during extinction in studies 1 and 2.

Conditioned predictors of interoceptive (ΔCS^{+}_{VISC}) and exteroceptive threats (**a**, study 1, ΔCS^{+}_{SOM}; **b**, study 2, ΔCS^{+}_{AUD}) induced shared differential activation in regions of interest (results of conjunction analyses, all P_{FWE}<0.05). Neural activations were superimposed on a structural T1-image and thresholded at P<0.001 uncorrected for visualization purposes; color bars indicate t-scores. For details, see Table S11.

Abbreviations: aINS, anterior insula; AUD, auditory; CS, conditioned stimuli; dACC, dorsal anterior cingulate cortex; dlPFC, dorsolateral prefrontal cortex; FWE, family-wise error; HIP, hippocampus; pINS, posterior insula; SOM, somatic; VISC, visceral; vmPFC, ventromedial prefrontal cortex.

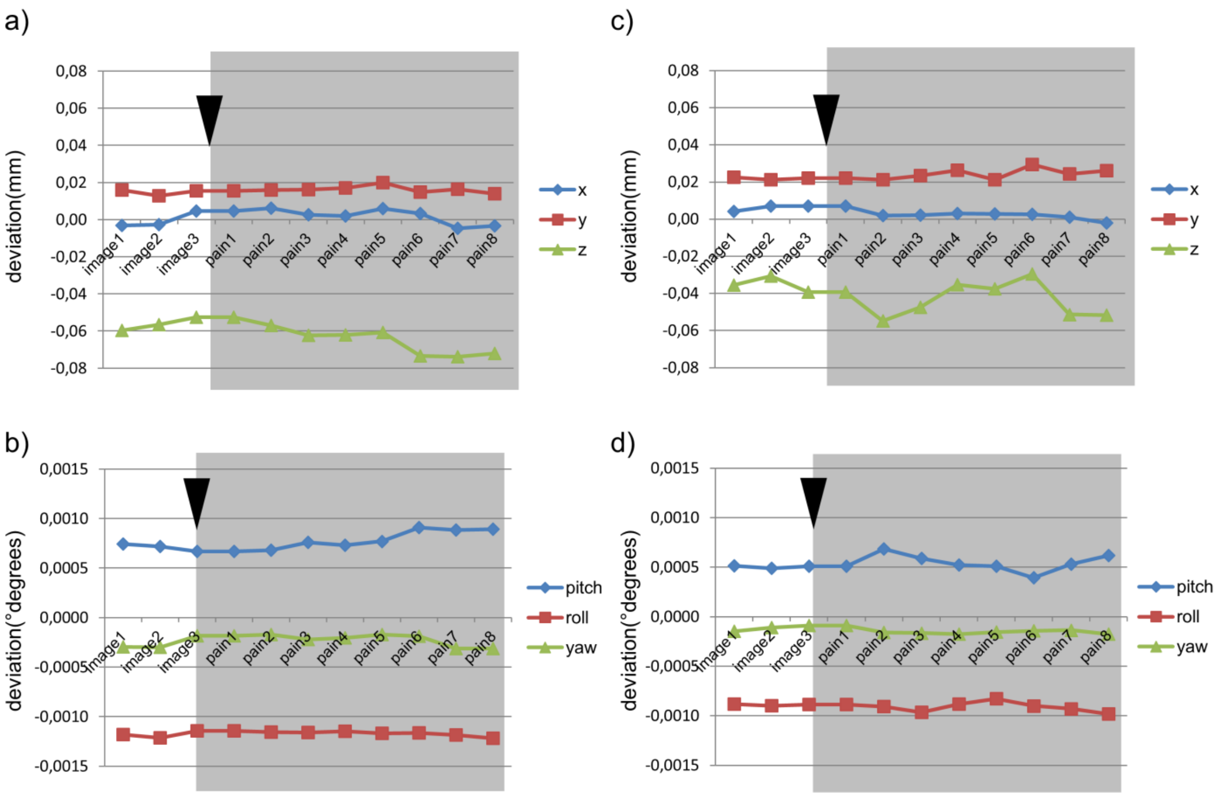

Figure S3: Head movement parameters in pain trials during habituation phase in study 1

In order to explore putative pain-modality specific head movements, head movement parameters (linear and degree movement, as extracted from the realignment procedure during preprocessing of MRI data) were analysed for the habituation phase in study 1 (N=42). This phase was accomplished after thresholding and matching, and prior to acquisition. Herein, five rectal distensions (visceral pain) and five heat pain stimuli (somatic pain) were delivered in pseudorandomized order. All pain stimuli were uncued, and stimulation intensities and durations were identical to those used in the subsequent acquisition phase when the same pain stimuli were implemented as unconditioned stimuli. Movement parameters were analysed for the three images (6.9 s) before visceral (**a**, **b**) or somatic (**c**, **d**) pain onset (indicated with black arrow), respectively, to create a baseline, and for eight images (18.4 s) after pain onset. Inspection of head movement timelines provided no indication of pain-induced rhythmical deviations after pain onsets in (**a**, **c**) line movement (mm) or (**b**, **d**) degree movement (°).

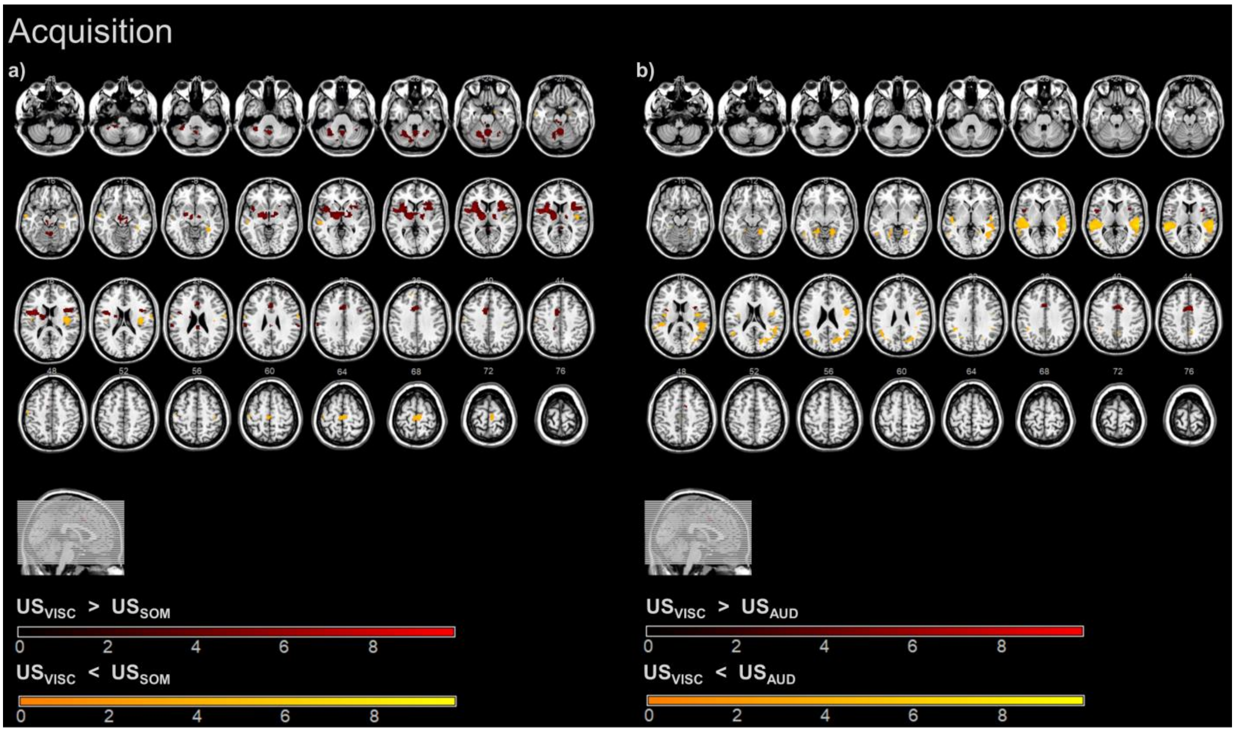

Figure S4: Results of whole-brain analyses of neural activation induced by interoceptive versus exteroceptive threats implemented as unconditioned stimuli (US) during acquisition in studies 1 and 2.

Neural activation induced by interoceptive threat (US_{VISC}) versus to exteroceptive threat implemented as US during acquisition (**a**, study 1, US_{SOM}; **b,** study 2, US_{AUD}; whole-brain analyses, $P_{uncorrected}$<0.001).
Neural activations were superimposed on a structural T1-image; color bars indicate t-scores. For details, see Figure 1, Table 1.
Abbreviations: AUD, auditory; SOM, somatic; US, unconditioned stimuli; VISC, visceral.

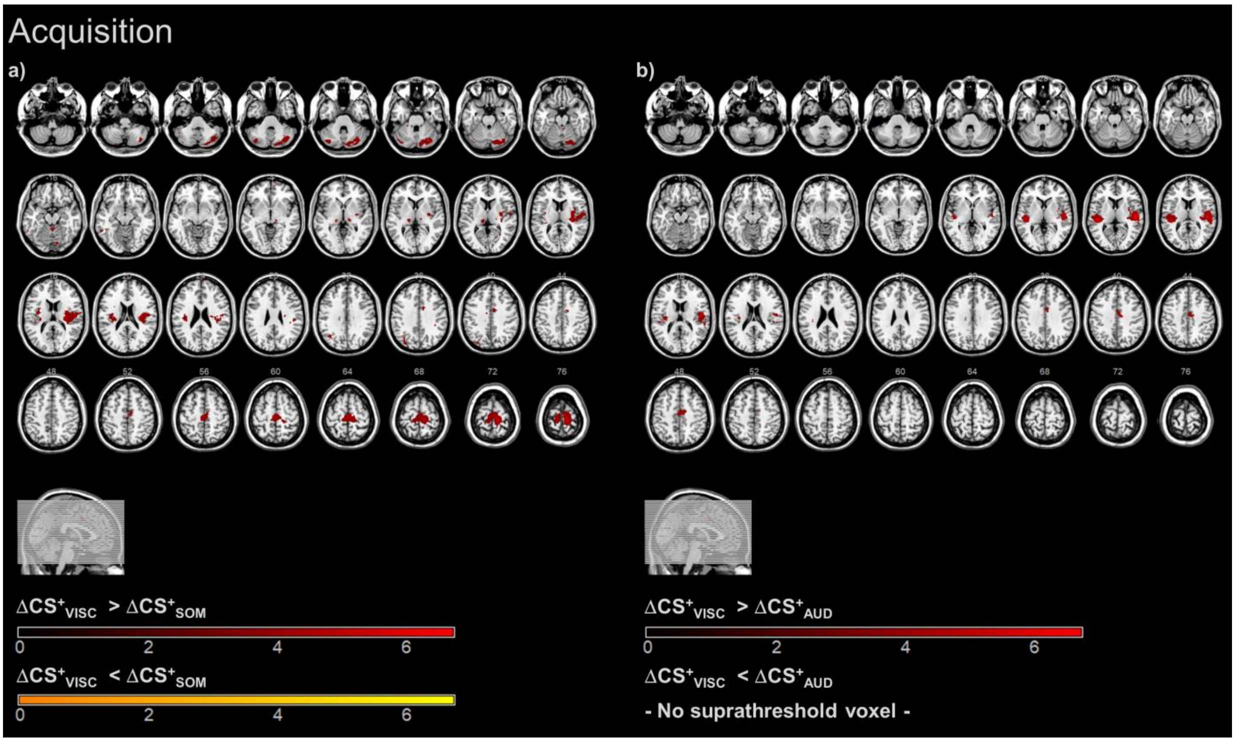

Figure S5: Results of whole-brain analyses of neural activation induced by cues (CS) predicting interoceptive and exteroceptive threats during acquisition in studies 1 and 2.

Neural activation induced by conditioned predictors of interoceptive threat (ΔCS^{+}_{VISC}) versus to exteroceptive threat implemented as CS during acquisition (**a**, study 1, ΔCS^{+}_{SOM}; **b,** study 2, ΔCS^{+}_{AUD}; whole-brain analyses, $P_{uncorrected}$<0.001).

Neural activations were superimposed on a structural T1-image; color bars indicate t-scores. For details, see Figure 2, Table 3.

Abbreviations: AUD, auditory; CS, conditioned stimuli; SOM, somatic; VISC, visceral.

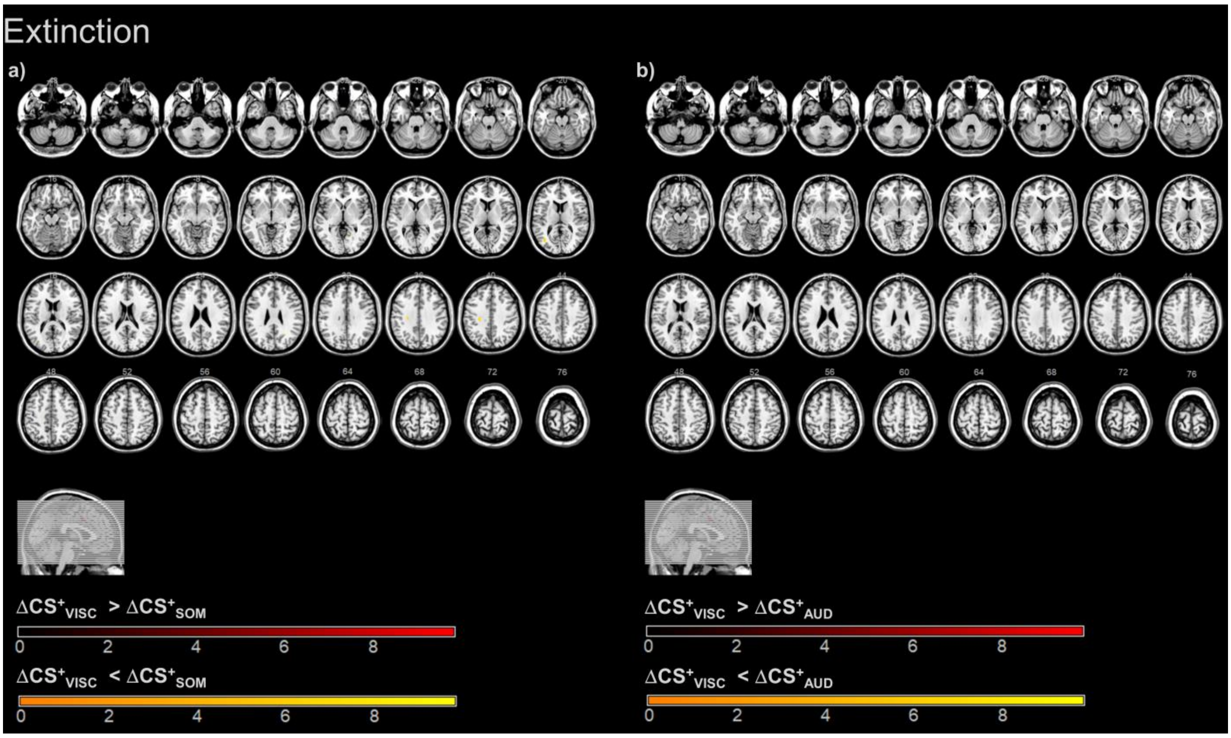

Figure S6: Results of whole-brain analyses of neural activation induced by cues (CS) predicting interoceptive and exteroceptive threats during extinction in studies 1 and 2.

Neural activation induced by conditioned predictors of interoceptive threat (ΔCS^{+}_{VISC}) versus to exteroceptive threat implemented as CS during extinction (**a**, study 1, ΔCS^{+}_{SOM}; **b,** study 2, ΔCS^{+}_{AUD}; whole-brain analyses, $P_{uncorrected}$<0.001).

Neural activations were superimposed on a structural T1-image; color bars indicate t-scores. For details, see Table S12.

Abbreviations: AUD, auditory; CS, conditioned stimuli; SOM, somatic; VISC, visceral.

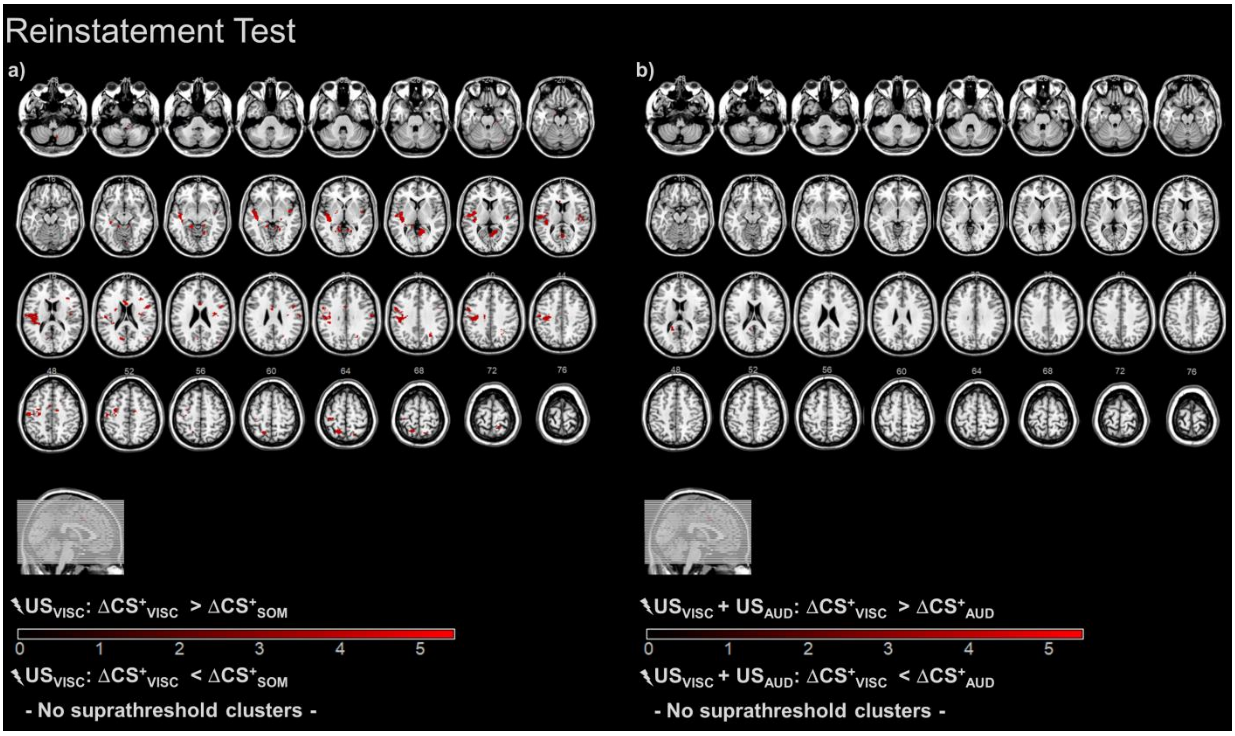

Figure S7: Results of whole-brain analyses of neural activation induced by cues (CS) predicting interoceptive and exteroceptive threats during reinstatement-test in studies 1 and 2.

Neural activation induced by conditioned predictors of interoceptive threat (ΔCS^{+}_{VISC}) versus to exteroceptive threat (study 1: ΔCS^{+}_{SOM}; study 2, ΔCS^{+}_{AUD}) implemented as conditioned stimuli (CS) during reinstatement-test (**a**, study 1: reinstatement with US_{VISC}; **b,** study 2: reinstatement with US_{VISC} and US_{AUD}; whole-brain analyses, $P_{uncorrected}$<0.001). Of note, no suprathreshold clusters were detectable during reinstatement-test with US_{SOM} ($P_{uncorrected}$<0.001). Neural activations were superimposed on a structural T1-image; color bars indicate t-scores. For details, see Figure 3, Table 4-6.
Abbreviations: AUD, auditory; CS, conditioned stimuli; SOM, somatic; US, unconditioned stimuli; VISC, visceral.

Supplementary analyses

For the acquisition phase, exploratory correlational analyses were performed for CS and US unpleasantness as well as CS- and US-induced neural activations based on extracted parameter estimate values. These analyses revealed that enhanced conditioned negative valence of interoceptive cues (ΔCS^{+}_{VISC}) correlated with greater negative valence of interoceptive threats (US_{VISC}) in both studies (all computed based on the differences between modalities for the respective CS and US in each study: r(40)=0.54; *P*<0.001 in study 1; r(21)=0.60; *P*=0.002 in study 2). This was paralleled by significant correlations between differential CS- and US-induced neural activation in some ROIs. Specifically, in study 1, differential neural activation induced by interoceptive threat predictors ($\Delta CS^{+}_{VISC} > \Delta CS^{+}_{SOM}$) in pINS was significantly associated with greater differential aINS activation induced by interoceptive threat ($US_{VISC} > US_{SOM}$) (r(40)=-0.34; *P*=0.030). Similar correlations were observed for study 2, revealing a correlation between enhanced interoceptive CS-induced activation ($\Delta CS^{+}_{VISC} > \Delta CS^{+}_{AUD}$) in MCC and enhanced differential interoceptive US-induced activation in dACC ($US_{VISC} > US_{AUD}$) (r(21)=0.52; *P*=0.010).

Abbreviations: aINS, anterior insula; AUD, auditory; CS, conditioned stimuli; dACC, dorsal anterior cingulate cortex; MCC, midcingulate cortex; pINS, posterior insula; ROI, regions of interest; SOM, somatic; US, unconditioned stimuli; VISC, visceral.

Supplementary references

1 Benson, S. *et al.* Cortisol affects pain sensitivity and pain-related emotional learning in experimental visceral but not somatic pain: a randomized controlled study in healthy men and women. *Pain* **160**, 1719-1728, doi:10.1097/j.pain.0000000000001579 (2019).

2 Koenen, L. R. *et al.* Greater fear of visceral pain contributes to differences between visceral and somatic pain in healthy women. *Pain*, doi:10.1097/j.pain.0000000000000924 (2017).

3 Koenen, L. R. *et al.* From anticipation to the experience of pain: The importance of visceral versus somatic pain modality in neural and behavioral responses to pain-predictive cues. *Psychosomatic medicine*, doi:10.1097/PSY.0000000000000612 (2018).

4 Mertz, H., Naliboff, B., Munakata, J., Niazi, N. & Mayer, E. A. Altered rectal perception is a biological marker of patients with irritable bowel syndrome. *Gastroenterology* **109**, 40-52 (1995).

5 Forkmann, K. *et al.* Pain-specific modulation of hippocampal activity and functional connectivity during visual encoding. *J Neurosci* **33**, 2571-2581, doi:10.1523/JNEUROSCI.2994-12.2013 (2013).

6 Forkmann, K., Wiech, K., Sommer, T. & Bingel, U. Reinstatement of pain-related brain activation during the recognition of neutral images previously paired with nociceptive stimuli. *Pain* **156**, 1501-1510, doi:10.1097/j.pain.0000000000000194 (2015).

7 Rolke, R. *et al.* Quantitative sensory testing: a comprehensive protocol for clinical trials. *Eur J Pain* **10**, 77-88, doi:10.1016/j.ejpain.2005.02.003 (2006).

8 Keszthelyi, D., Troost, F. J. & Masclee, A. A. Irritable bowel syndrome: methods, mechanisms, and pathophysiology. Methods to assess visceral hypersensitivity in irritable bowel syndrome. *Am J Physiol Gastrointest Liver Physiol* **303**, G141-154, doi:10.1152/ajpgi.00060.2012 (2012).

9 Boucsein, W. *et al.* Publication recommendations for electrodermal measurements. *Psychophysiology* **49**, 1017-1034, doi:10.1111/j.1469-8986.2012.01384.x (2012).

10 Pineles, S. L., Orr, M. R. & Orr, S. P. An alternative scoring method for skin conductance responding in a differential fear conditioning paradigm with a long-duration conditioned stimulus. *Psychophysiology* **46**, 984-995, doi:10.1111/j.1469-8986.2009.00852.x (2009).

11 Allen, M., Poggiali, D., Whitaker, K., Marshall, T. R. & Kievit, R. A. Raincloud plots: a multi-platform tool for robust data visualization. *Wellcome Open Res* **4**, 63, doi:10.12688/wellcomeopenres.15191.1 (2019).
