# Supplementary material for: Associative learning and extinction of conditioned threat predictors across sensory modalities
Source: Commun Biol. 2021 May 11;4:553. doi: 10.1038/s42003-021-02008-1 (PMC8113515; doi:10.1038/s42003-021-02008-1)
Supplement: Supplementary file 3 — Reporting Summary [file 42003_2021_2008_MOESM3_ESM.pdf]

natureresearch

Corresponding author(s): Prof. Dr. Sigrid Elsenbruch

Last updated by author(s): Mar 2, 2021

Reporting Summary

Nature Research wishes to improve the reproducibility of the work that we publish. This form provides structure for consistency and transparency in reporting. For further information on Nature Research policies, see Authors & Referees and the Editorial Policy Checklist .

Statistics

For all statistical analyses, confirm that the following items are present in the figure legend, table legend, main text, or Methods section.

n/a	Confirmed	
☐	☒	The exact sample size (*n*) for each experimental group/condition, given as a discrete number and unit of measurement
☐	☒	A statement on whether measurements were taken from distinct samples or whether the same sample was measured repeatedly
☐	☒	The statistical test(s) used AND whether they are one- or two-sided<br>*Only common tests should be described solely by name; describe more complex techniques in the Methods section.*
☐	☒	A description of all covariates tested
☐	☒	A description of any assumptions or corrections, such as tests of normality and adjustment for multiple comparisons
☐	☒	A full description of the statistical parameters including central tendency (e.g. means) or other basic estimates (e.g. regression coefficient) AND variation (e.g. standard deviation) or associated estimates of uncertainty (e.g. confidence intervals)
☐	☒	For null hypothesis testing, the test statistic (e.g. *F*, *t*, *r*) with confidence intervals, effect sizes, degrees of freedom and *P* value noted<br>*Give P values as exact values whenever suitable.*
☒	☐	For Bayesian analysis, information on the choice of priors and Markov chain Monte Carlo settings
☒	☐	For hierarchical and complex designs, identification of the appropriate level for tests and full reporting of outcomes
☐	☒	Estimates of effect sizes (e.g. Cohen's *d*, Pearson's *r*), indicating how they were calculated

Our web collection on statistics for biologists contains articles on many of the points above.

Software and code

Policy information about availability of computer code

Data collection	Presentation (19.0 Build 02.27.17); software thermal device (Pathway, pain and sensory evaluation system, Arbel 6.4.0.26.3, 2005, Medoc Ltd.); software barostat system (protocol plus 6.7R, 2004, G&J Electronics); software sound system (Audacity 1.3.10-beta)<br>Electrodermal activity (EDA) was recorded using an MRI-compatible system (Biopac Systems, Inc., Goleta, CA, USA; MP100 in study 1, MP160 in study 2)
Data analysis	IBM SPSS Statistics 20 (IBM Corp., Armonk, N.Y., USA); SPM 12 (SPM12, Wellcome Trust Centre for Neuroimaging, UCL, London, UK); Microsoft Excel 2010; Matlab R2016b (R2016b, Mathworks Inc., Sherborn, MA, USA)<br>AcqKnowledge Software (Biopac; Version 3.9 in study 1, Version 4.3 in study 2)

For manuscripts utilizing custom algorithms or software that are central to the research but not yet described in published literature, software must be made available to editors/reviewers. We strongly encourage code deposition in a community repository (e.g. GitHub). See the Nature Research guidelines for submitting code & software for further information.

Data

Policy information about availability of data

All manuscripts must include a data availability statement. This statement should provide the following information, where applicable:

- Accession codes, unique identifiers, or web links for publicly available datasets
- A list of figures that have associated raw data
- A description of any restrictions on data availability

All fMRI data analysed for the current study are available in the neurovault repository (https://neurovault.org/collections/GPPGVZAT/). Behavioural and EDA data are provided in the main manuscript or its supplementary materials.

Field-specific reporting

Please select the one below that is the best fit for your research. If you are not sure, read the appropriate sections before making your selection.

☐ Life sciences ☒ Behavioural & social sciences ☐ Ecological, evolutionary & environmental sciences

For a reference copy of the document with all sections, see nature.com/documents/nr-reporting-summary-flat.pdf

Behavioural & social sciences study design

All studies must disclose on these points even when the disclosure is negative.

Study description	Data from two separate, yet complementary differential fear conditioning studies are reported, providing quantitative, experimental behavioral and neural (BOLD) data.
Research sample	Healthy volunteers were recruited by local advertisement for both studies. Data are reported from N=42 volunteers for study 1 (all female, age 34.5±2.0 years; BMI 22.7±0.4 kg/m²), and N=23 volunteers for study 2 (10 female, age 26.7±1.0 years; BMI 22.4±0.7 kg/m²).
Sampling strategy	Sample sizes for both studies were chosen based on he established criteria for valid and replicable BOLD data (Cremers, Wager & Yarkoni, Plos One (2017)), reporting a positive correlation between statistical power and sample size (power coefficient of 1.0 for N>40 and 0.8 for N>20).
Data collection	Questionnaire battery (pen & paper); visual analogue scales during fMRI scanning: response device in the MRI scanner; barostat system (modified ISOBAR 3 device, G & J Electronics, Toronto, ON, Canada); thermal device (PATHWAY model CHEPS; Medoc Ltd. Advanced Medical Systems, Ramat Yishai, Israel); auditory system (Amplifier mkll+S/N 2016-2-2-03); Electrodermal activity (EDA) was recorded online from electrodes placed on the thenar and hypothenar of the left hand using an MRI-compatible system (Biopac Systems, Inc., Goleta, CA, USA; MP100 in study 1, MP160 in study 2).<br>during data collection, medical staff was present for medical examination prior to testing and to ensure the well-being of participants during the experiment; experimenters were not blinded to the studies hypotheses
Timing	Data collection was from July 2015 until February 2019 in study 1 and from December 2017 - November 2018 in study 2
Data exclusions	From the total sample of N=77, N=12 were excluded due to technical difficulties with data acquisition (N=6), movement artefacts (N=4), or failure to reach visceral pain threshold within predetermined maximal distension pressure (N=2), resulting in a total sample of N=65 (N=42 in study 1 and N=23 in study 2).
Non-participation	No participants dropped out/ declined participation
Randomization	Participants were not allocated to experimental groups, except for the different reinstatement procedures in study 1, to which participants were pseudorandomly assigned prior to the experiment.

Reporting for specific materials, systems and methods

We require information from authors about some types of materials, experimental systems and methods used in many studies. Here, indicate whether each material, system or method listed is relevant to your study. If you are not sure if a list item applies to your research, read the appropriate section before selecting a response.

Materials & experimental systems

n/a	Involved in the study	
☒	☐	Antibodies
☒	☐	Eukaryotic cell lines
☒	☐	Palaeontology
☒	☐	Animals and other organisms
☐	☒	Human research participants
☒	☐	Clinical data

Methods

n/a	Involved in the study	
☒	☐	ChIP-seq
☒	☐	Flow cytometry
☐	☒	MRI-based neuroimaging

Human research participants

Policy information about [studies involving human research participants]

Population characteristics	See above.
Recruitment	Participants were recruited via local advertisement for both studies.
Ethics oversight	The work was conducted in accordance with the Declaration of Helsinki, and studies were approved by the ethics committee of the University Hospital Essen (protocol numbers 10-4493, 16-7237), and followed the relevant ethical guidelines and regulations.

Note that full information on the approval of the study protocol must also be provided in the manuscript.

Magnetic resonance imaging

Experimental design

Design type

Event-related fMRI, task-based (differential delay conditioning) during three different experimental phases (acqusition, extinction and reinstatement test)

Design specifications

Regressors chosen per experimental session (acquisition, extinction, reinstatement (session included in the model, data not reported) reinstatement test) for both studies:
CS type (CS+VISC; CS+SOM/CS+AUD; CS-) and US modality (USVISC; USSOM/USAUD, analyses of acquisition phases only).

Acquisition: study 1: 10 per CS; 8 per US; study 2: 12 per CS, 10 per US. Duration CS: 6-12s (jittered, exact stimulus durations were used); duration US - ascending and plateau phases: (study 1: both US 20 s; study 2: both US 14 s)
Extinction: study 1: 5 per CS; study 2: 12 per CS
Reinstatement: study 1: 4 per US; study 2: 3 per US
Reinstatement test: study 1: 5 per CS; study 2: 12 per CS
Movement parameters (6 per subject/session).

Behavioral performance measures

All participants were asked to rate CS and US valence as well as CS-US contingencies on visual analogue scales (VAS) in both studies. Additionally US intensity ratings were obtained in study 1.

All VAS were rated on digitized VAS using a MRI-compatible handheld device within the scanning room (computer mouse) and ratings were monitored by experimenters during the fMRI scanning procedure from outside of the scanning room to ensure participants were awake.

Acquisition

Imaging type(s)

Structural and functional MRI

Field strength

3 Tesla

Sequence & imaging parameters

Structural imaging: T1-weighted 3D-MPRAGE sequence (TR 1900ms, TE 2.13ms, flip angle 9°, FOV 239 x 239mm2, 192 slices, slice thickness 0.9mm, voxel size 0.9 x 0.9 x 0.9mm3, matrix 256 x 256mm2, GRAPPA r = 2).

Functional imaging: single-shot echo-planar imaging (EPI) sequences (TE 28.0ms, flip angle 90°, GRAPPA r = 2 with 38 transversal slices angulated in the direction of the corpus callosum, slice thickness of 3mm, slice gap 0.6mm, voxel size 2.3 x 2.3 x 3.0mm; study 1: TR 2300ms, FOV 220 x 220mm2, matrix 94 x 94mm2; study 2: TR 2400ms, FOV 240 x 240mm2, matrix 104 x 104mm2).

Area of acquisition

whole-brain scan

Diffusion MRI ☐ Used ☒ Not used

Preprocessing

Preprocessing software

SPM12 (Wellcome Trust Centre for Neuroimaging, UCL, London, UK) implemented in Matlab R2016b (Mathworks Inc., Sherborn, MA, USA); For data preprocessing Realignement, Co-registration, Normalization, and Smoothing procedures were used. For realignement and normalization parameters see below. No segmentation was performed. Co-registration: Functional images were co-registered to individual T1-weighted structural images used as reference images, with the origin set to the anterior commissure. All functional images were smoothed using an isotropic Gaussian kernel of 8mm. To correct for low frequency drifts, a temporal high-pass filter with a cut-off set at 128s was implemented.

Normalization

Linear transformation to Montreal Neurological Institute (MNI) space

Normalization template	Standardized ICBM template for European brains as implemented in SPM12
Noise and artifact removal	Realignment procedure (six motion parameters for translation (x, y, z) and for rotation (pitch, roll, yaw))
Volume censoring	none

Statistical modeling & inference

Model type and settings

For the first level, statistical analyses were performed using a mass univariate approach based on General linear models applied to the EPI images. The time series of each voxel was fitted with a corresponding task regressor that modelled a box car convolved with a canonical hemodynamic response function (HRF). For the first level, a fixed effects model was used (units: secs; TR: 2.3 for study 1; 2.5 for study 2) regressors as in design specification (see above)); For the second level a random effects model was chosen (effects see "effects tested", below).
Serial autocorrelations were taken into consideration by means of an autoregressive model first-order correction.

Effect(s) tested

First-Level: For each regressor (CS/US) and combination of regressors per stimulus (CS/US) and experimental phases, simple and differential T-contrasts were computed.
Second-level: For analyses of differential neural activation, one-sample t-tests were calculated based on differential first-level contrasts. For conjunction analyses against global and conjunction null, paired-T-tests were calculated.

Specify type of analysis: ☐ Whole brain ☒ ROI-based ☐ Both

Anatomical location(s)

ROIs were defined based on the Automated Anatomical Labeling atlas. Segmentation of the insula (aINS, pINS) and cingulate cortex (dACC, MCC) was accomplished with masks based on previous literature (Deen et al., 2011) within the borders of the WFU Pick Atlas.

Statistic type for inference
(See Eklund et al. 2016)

T- and P(FWE)-and P(uncorrected)-values are reported for all significant peak voxel within a priori defined ROI (voxel-wise statistics).

Correction

For all reported ROI-analyses, familywise-error (FWE) correction for multiple testing was used with statistical significance set at pFWE < 0.05, and coordinates refer to the MNI space. For additional whole brain analyses, uncorrected results (p<0.001) are reported.

Models & analysis

n/a	Involved in the study
☒	☐ Functional and/or effective connectivity
☒	☐ Graph analysis
☒	☐ Multivariate modeling or predictive analysis
